# MASLD is related to impaired alcohol dehydrogenase (ADH) activity and elevated blood ethanol levels: Role of TNFα and JNK

**DOI:** 10.1016/j.redox.2024.103121

**Published:** 2024-03-12

**Authors:** Katharina Burger, Finn Jung, Katharina Staufer, Ruth Ladurner, Michael Trauner, Anja Baumann, Annette Brandt, Ina Bergheim

**Affiliations:** a Department of Nutritional Sciences, Molecular Nutritional Science, University of Vienna, Vienna, Austria; b Department of Internal Medicine III, Division of Gastroenterology & Hepatology, Medical University of Vienna, Vienna, Austria; c Department of Surgery, Division of Transplantation, Medical University of Vienna, Vienna, Austria; d Department of General, Visceral and Transplant Surgery, Eberhard-Karls-University Tuebingen, Tuebingen, Germany

**Keywords:** Alcohol dehydrogenase, Alcohol metabolism, c-Jun N-terminal kinase, Tumor necrosis factor alpha, Steatotic liver disease

## Abstract

Elevated fasting ethanol levels in peripheral blood frequently found in metabolic dysfunction-associated steatohepatitis (MASLD) patients even in the absence of alcohol consumption are discussed to contribute to disease development. To test the hypothesis that besides an enhanced gastrointestinal synthesis a diminished alcohol elimination through alcohol dehydrogenase (ADH) may also be critical herein, we determined fasting ethanol levels and ADH activity in livers and blood of MASLD patients and in wild-type ± anti-TNFα antibody (infliximab) treated and TNFα^-/-^ mice fed a MASLD-inducing diet. Blood ethanol levels were significantly higher in patients and wild-type mice with MASLD while relative ADH activity in blood and liver tissue was significantly lower compared to controls. Both alterations were significantly attenuated in MASLD diet-fed TNFα^-/-^ mice and wild-type mice treated with infliximab. Moreover, alcohol elimination was significantly impaired in mice with MASLD. In *in vitro* models, TNFα but not IL-1β or IL-6 significantly decreased ADH activity. Our data suggest that elevated ethanol levels in MASLD patients are related to TNFα-dependent impairments of ADH activity.

## Highlights

• Blood alcohol levels are elevated in humans and mice with MASLD.
• Activity of ADH in blood and liver tissue is lower in patients with MASLD.
• Alcohol clearance is impaired in mice with early signs of MASLD.
• Impairments of ADH activity are related to an activation of JNK signaling and serine phosphorylation of ADH.

## Graphical abstract

**Figure:**
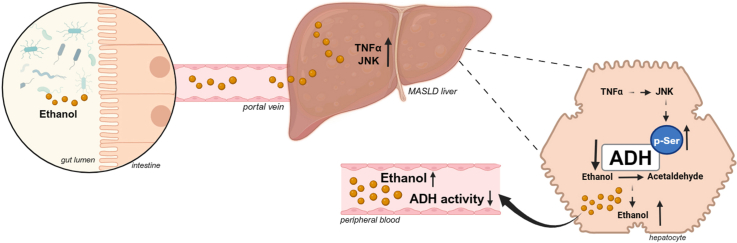

## 1 Summary “box”

### 1.1 What is already known about this subject?

• In humans and ob/ob mice, the development of metabolic dysfunction-associated steatotic liver disease (MASLD) is associated with elevated ethanol levels in the absence of alcohol consumption.
• Intestinal microbiota can produce ethanol.
• Activity of alcohol dehydrogenase (ADH) in liver tissue of ob/ob mice is lower than in lean mice.

### 1.2 What are the new findings?

• Activity of ADH in blood and liver tissue is lower in patients with MASLD.
• Alcohol clearance is impaired in mice with early signs of MASLD.
• TNFα-dependent impairments of ADH activity are related to an activation of c-Jun N-terminal kinase (JNK) signaling and serine phosphorylation of ADH.
• TNFα^-/-^ mice are protected from the loss of hepatic ADH activity and elevated endogenous ethanol levels.
• Treatment with the anti-TNFα antibody infliximab attenuates the increase in endogenous ethanol levels and decrease of hepatic ADH activity in mice with MASLD.

## 2 Introduction

Metabolic dysfunction-associated steatotic liver disease (MASLD) is by now the most common liver disease world-wide affecting ∼38 % of the global population [1]. Insulin resistance has been defined as the key risk factor for the development of MASLD often being associated with overweight or even obesity [2]. In the last 10-15 years, results of studies suggest that not only a general overnutrition along with several single nucleotide polymorphisms may be critical in the development of MASLD. Rather, specific dietary patterns like the so called “Westernized diet” being rich in saturated fatty acids, cholesterol and sugar but low in fiber may also be critical [3]. Furthermore, changes in intestinal microbiota and barrier function being associated with an increased permeation of bacterial endotoxins and an induction of Toll-like receptors (TLRs) in liver tissue and elevated ethanol levels in peripheral blood have also been associated even in the absence of alcohol intake with the development of MASLD [4,5]. Especially the role of the latter in the development of MASLD and the underlying molecular mechanisms have not yet been fully understood.

Already more than a decade ago, it was first reported that both, patients with MASLD and ob/ob mice with MASLD, show higher fasting blood and breath ethanol levels than lean controls despite no ethanol intake before obtaining blood samples [[6], [7], [8]]. Results of recent years suggest that the increased ethanol concentrations in blood and breath of adult and pediatric patients with MASLD as well as mice with MASLD may be related to changes of fecal microbiota composition leading subsequently to an increased formation of ethanol by the intestinal microbiota [9,10]. Indeed, it recently has been shown that following a mixed-meal, ethanol levels in peripheral blood are significantly increased in patients with MASLD and metabolic dysfunction-associated steatohepatitis (MASH), the latter showing even higher levels [10]. A treatment with the alcohol dehydrogenase (ADH) inhibitor 4-methylpyrazole enhanced this effect of ingesting food, while a concomitant treatment with antibiotics almost completely abolished the increase of ethanol in peripheral blood [10]. Results of our own group and others suggest that not only an increased endogenous synthesis of ethanol may lead to an elevation of fasting ethanol in MASLD patients, but that herein alterations of ADH in liver tissue may be critical, too [4,11]. In line with these findings, others have reported before that the activity of ADH in liver tissue is lower in diabetic rats than in healthy control animals [12]. Results of *in vivo* studies in mice of our own group and *in vitro* studies of others further suggest that activity of ADH in liver might be modulated through tumor necrosis factor alpha (TNFα) and insulin-dependent signaling cascades [4,13]. However, molecular mechanisms underlying the regulation of ADH and if these alterations in ADH activity are also found in patients with MASLD have not yet been clarified.

Starting from this background, the first aim of the present study was to determine if the elevated ethanol levels in fasted patients with MASLD are related to impairments of ADH activity. The second aim of the study was then to determine if these alterations are related to proinflammatory cytokines and herein especially TNFα.

## 3 Methods

### 3.1 Human studies

All procedures were approved by the ethics committee of the Tuebingen University Hospital, Tuebingen, Germany (510/2017BO2), and the Medical University of Vienna, Austria (747/2011). All subjects gave written informed consent. Patients & controls study I: A total of 10 patients with MASLD and 8 age-matched non-MASLD patients (=control) undergoing partial liver resection (e.g., resection for liver metastasis) were enrolled (also see Ref. [14] for further details). ADH activity in plasma was only measured in 7 disease-free and 7 MASLD patients due to a lack of sample. MASLD was diagnosed according to EASL criteria for non-alcoholic fatty liver disease (NAFLD) considering liver histology [15]. Liver tissue and plasma obtained from the subjects were stored in liquid nitrogen and at -80 °C. Patients & controls study II: 16 healthy age-matched controls, 20 patients with simple steatosis and 44 patients with MASH diagnosed by ultrasound and/or liver biopsy according to EASL criteria for NAFLD considering liver histology [15] were enrolled in this study and serum was collected. While ethanol levels were measurable in all samples, ADH activity was only measurable in 13 controls, 18 patients with steatosis and 34 patients with MASH due to a lack of sample. Characteristics of patients and their respective controls of study I and II are summarized in Table 1, Table 2.

**Table 1:** Characteristics of patients with and without MASLD undergoing liver resection (Patients & controls study I).

Parameter	Control^#^	MASLD
**n**	8	10
**Sex (f/m)**	4/4	3/7
**Age (years)**	54.1 ± 6.3	63.4 ± 2.5
**Height (m)**	1.7 ± 0.0	1.7 ± 0.0
**Weight (kg)**	67.0 ± 4.3	80.8 ± 3.4*
**BMI (kg/m**^**2**^**)**	22.5 ± 1.0	26.6 ± 0.9*
**AST (U/l)**	35.3 ± 9.4	38.9 ± 6.6
**ALT (U/l)**	34.0 ± 7.9	51.1 ± 13.6
**γ-GT (U/l)**	49.8 ± 14.8	150.2 ± 30.0*

Data are shown as total numbers and means ± SEM, n = 8 controls except for ALT: n = 7 controls as values were not assessed and n = 10 MASLD patients. ^#^controls are non-MASLD patients undergoing liver resection; *p≤0.05 compared to controls calculated by Student's *t*-test; BMI, body mass index; AST, aspartate aminotransferase; ALT, alanine aminotransferase; γ-GT, gamma-glutamyltransferase.

**Table 2:** Characteristics of patients with biopsy proven MASLD and controls (Patients & controls study II).

Parameter	Control	Steatosis	MASH
n	16	20	44
**Sex (f/m)**	7/9	10/10	26/18
**Age (years)**	41.8 ± 3.1	44.2 ± 3.3	47.4 ± 2.3
**Height (m)**	1.7 ± 0.0	1.7 ± 0.0	1.7 ± 0.0
**Weight (kg)**	67.1 ± 3.2	94.4 ± 5.2*	111.3 ± 4.6*^†^
**BMI (kg/m**^**2**^**)**	22.0 ± 0.6	33.3 ± 2.5*	38.3 ± 1.4*
**AST (U/l)**	23.8 ± 1.5	30.0 ± 2.3	42.6 ± 4.3*
**ALT (U/l)**	20.3 ± 2.1	43.9 ± 6.1*	52.2 ± 5.7*
**γ-GT (U/l)**	16.3 ± 1.3	95.6 ± 25.7*	72.0 ± 10.9*
**TG (mg/dl)**	72.4 ± 5.3	170.1 ± 18.3*	168.6 ± 14.5*
**Fasting glucose (mg/dl)**	85.8 ± 1.7	108.6 ± 10.3*	109.5 ± 4.7*
**Insulin (mU/l)**	5.7 ± 0.5	15.3 ± 3.2*	23.4 ± 2.4*
**HOMA-IR**	1.2 ± 0.1	4.6 ± 1.2*	6.3 ± 0.7*

Data are shown as total numbers and means ± SEM, n = 16 controls, n = 20 patients with steatosis and n = 44 patients with MASH except for insulin: steatosis n = 17, MASH n = 35 as values were not assessed; *p≤0.05 compared to controls and ^†^p≤0.05 compared to steatosis calculated by one-way ANOVA followed by Tukey's post-hoc test. BMI, body mass index; AST, aspartate aminotransferase; ALT, alanine aminotransferase; HOMA-IR, homeostasis model assessment index; γ-GT, gamma-glutamyltransferase; TG, triglycerides.

### 3.2 Mouse experiments

Mice in all experiments were housed in specific-pathogen-free barrier facility accredited by the Association for Assessment and Accreditation of Laboratory Animal care. All experiments were carried out under controlled conditions and mice had free access to tap water at all times (12h/12h light/dark cycle, 23 °C, 65 % relative humidity). All procedures were approved by the local institutional animal care and use committee (Federal Ministry of Austria Education, Science and Research, Austria; BMWFW-66.006/0020-WF/V/3b/2017; 2020-0.702.135; 2023-0.284.375) and animals were handled in accordance to the European Convention for the Protection of Vertebrate Animals used for Experimental and Other Scientific Purposes and experiments were carried out in compliance with the ARRIVE guidelines. In a first set of experiments, six to eight weeks old male C57BL/6J mice (Janvier SAS, Le Genest-Saint-Isle, France) (n = 6–7/group) were fed either a liquid control diet (C, 69 E% carbohydrates, 12 E% fat, 19 E% protein) or a liquid sucrose-, fat-, and cholesterol-rich diet (SFC, 55 E% carbohydrates, 30 E% fat derived from butterfat, 15 E% protein with 50% (wt./wt.) sucrose and 0.16% (wt./wt.) cholesterol; Ssniff, Soest, Germany) for eight weeks after adaption to the liquid control diet as detailed previously [16]. In a second set of experiments, six to eight weeks old male C57BL/6J mice (Janvier SAS, Le Genest-Saint-Isle, France) were also either fed a pelleted SFC or standard pelleted chow (pC, 69 E% carbohydrates, 18 E% protein and 13 E% fat; pSFC, 43 E% carbohydrates, 15 E% protein and 42 E% fat; Ssniff) *ad libitum* for eight weeks and received then once a gavage with ethanol (3.5 mg/g bw) (n = 3–4/group). In a third set of experiments, six to eight weeks old male C57BL/6J (Jackson Laboratory, Bar Harbor, ME, USA) and TNFα^-/-^ mice (n = 6–8/group) (B6.129S-Tnftm1Gkl/J, Jackson Laboratory, Bar Harbor, ME, USA) from own breeding were either fed the SFC or the C diet for nine weeks after adaption to the liquid control diet. Dietary intake was assessed daily, and bodyweight was measured weekly. Further information regarding feeding and study design has been described in detail previously [17]. In a fourth set of experiments six to eight weeks old male C57BL/6J mice (Janvier SAS, Le Genest-Saint-Isle, France) were either pair-fed the SFC or the C diet for seven weeks and were then randomly assigned to either be treated three times/week intraperitoneally (i.p.) with 10 mg/kg bw infliximab (anti-TNFα antibody (Sigma-Aldrich, Steinheim, Germany)) or vehicle (0.9 % NaCl) for one week (n = 6–8/group). Further information regarding feeding and study design has been described in detail previously [17]. A glucose tolerance test (GTT) was performed in the first, the second and the third set of experiments one week prior sacrificing. Mice were fasted for 6 h and after assessing fasting blood glucose concentrations, in experiments 1 and 3 a glucose solution (2 mg/kg bw) was injected i.p. while in experiment 2 mice received the glucose solution (2 mg/kg bw) per oral gavage. In all GTTs, blood was collected from the tail vein to assess blood glucose concentrations with a standard glucometer (Contour, Bayer Vital GmbH, Leverkusen, Germany). At the end of all four trials mice were anesthetized with a ketamine/xylazine mixture (100 mg ketamine/kg bw; 16 mg xylazine/kg bw) with an i.p. injection and killed by cervical dislocation. Blood was collected from portal vein and vena cava. Liver and intestinal tissue samples were collected and snap-frozen or fixed in neutral-buffered formalin. Study designs are summarized in Fig. 2A and F, Fig. 4, Fig. 5A and in case of study 3 and 4, study designs have also been described in detail previously [17].

### 3.3 Cell culture

Alpha mouse liver 12 (AML-12) cells, isolated hepatocytes from the normal liver of a 3 month old mouse obtained from American type culture collection (ATCC CRL-2254; ATCC, VA, USA) were grown in DMEM/F12 media (PAN-Biotech, Aidenbach, Germany) supplemented with 10 % fetal bovine serum (FBS), 40 ng/ml dexamethasone, 0.005 mg/ml insulin, 5 ng/ml selenium and 0.005 mg/ml transferrin (PAA Laboratories, Colbe, Germany), as well as penicillin and streptomycin (1 %, PAN-Biotech, Aidenbach, Germany) at humidified 37 °C and 5 % carbon dioxide. After starvation for 18 h in media supplemented with FBS (0.01 %), cells were challenged with either TNFα (10 and 20 ng/ml), interleukin (IL)-1β (10 and 20 ng/ml) or IL-6 (10 and 20 ng/ml) (#315-01A; #211-11B; #216-16; Thermo Fisher Scientific, MA, USA) for 6 h. Concentrations used in the cell culture experiment were chosen based on previous studies of us and others assessing the effects of TNFα, IL-1β and IL-6 in mouse hepatocytes [4,18]. For another experiment cells were preincubated with a c-Jun N-terminal kinase (JNK) antagonist (50 μM) (SP600125, Thermo Fisher Scientific, MA, USA) and a JNK agonist (1 pg/ml) (Anisomycin, Biomol GmbH, Hamburg, Germany) for 1 h and were then challenged with TNFα (10 ng/ml) for 6 h. Cells were trypsinized and ADH activity was measured in the cytosolic fractions as detailed below. Study design is summarized in Fig. 3A.

### 3.4 Isolation of cytosolic and mitochondrial fractions as well as measurement of ADH and ALDH2 activity

ADH activity was measured in cytosol of mouse and human liver, in the cytosolic fraction of AML-12 cells and in peripheral serum and plasma, respectively, using the method previously described by Ming et al. [19]. Acetaldehyde dehydrogenase 2 (ALDH2) activity in the mitochondrial fraction of mouse and human livers was determined using a commercial kit following the protocol of the manufacturer (#E4587; BioVision, MA, USA). For the isolation of mitochondrial and cytosolic fraction liver tissue was homogenized in 1.15 % ice cold potassium chloride and fractionated by ultracentrifugation (100 000 g for 1 h). Cells were homogenized and sonicated for 5 min and then centrifuged by ultracentrifugation (43 000 g for 1 h) for isolation of the cytosolic fraction. The enzymatic kinetic measurement for ADH activity was immediately conducted and changes in extinction at 340 nm for 1 h at 37 °C were measured. All measurements in the cytosolic and mitochondrial fraction of liver tissue were normalized to the cell number (determined with DNA concentrations by measuring absorbance (260/280 nm) in the nuclear fraction of the cells), in the cytosolic fraction of AML-12 cells to the whole protein concentration (determined with Bradford assay) and in serum/plasma to the amount of ADH1 protein determined by Western Blot (see below).

### 3.5 Evaluation of liver damage and inflammation

Paraffin-embedded liver sections (4 μm) were stained with hematoxylin and eosin (H&E) (Sigma Aldrich Chemie GmbH, Steinheim, Germany) to evaluate liver histology using the NAFLD activity score (NAS) as detailed by Kleiner et al. [20]. Representative pictures were captured using a microscope integrated camera (LeicaDM4000 B LED, Leica, Wetzlar, Germany). Alanine aminotransferase (ALT) activity in murine plasma was measured in a routine laboratory (Veterinary Medical University of Vienna, Vienna, Austria).

### 3.6 Ethanol levels in plasma, serum, and small intestine

Ethanol levels in human and murine plasma samples and human serum as well as in mouse duodenal lumen were measured using a commercially available kit (Ethanol FS, Diagnostic Systems GmbH, Holzheim, Germany).

### 3.7 Western Blot

Proteins (0.05 μg/μl for ADH1 and 4, 1 μg/μl for β-actin and 0.1 μg/μl for ALDH2 in liver, 5 μg/μl for ADH1 in plasma) were separated on 10 % SDS-polyacrylamide gels and transferred to polyvinylidene difluoride membranes (Bio-Rad Laboratories, Hercules, California, USA). Membranes were incubated with specific primary antibodies (#5295 ADH1, #18818 ALDH2, Cell Signaling Technology, Massachusetts, USA; sc-515217 ADH4, sc-47778 β-actin; Santa Cruz Biotechnology, TX, USA) and respective secondary antibodies (#7074 anti-rabbit IgG, HRP-linked, #7076 anti-mouse IgG, HRP-linked; Cell Signaling Technology, MA, USA). To detect the protein bands, the Super Signal West Dura kit (Thermo Fisher Scientific, MA, USA) was used, and densitometric analysis was performed using ChemiDoc XRS System (Bio-Rad Laboratories, Hercules, CA, USA) as detailed previously [16].

### 3.8 Immunoprecipitation of ADH1

To determine phosphorylation of ADH1, an immunoprecipitation of ADH1 in AML-12 cells using a commercially available kit was performed (Pierce^TM^ Classic IP Kit (#26146, Sigma-Aldrich Chemie, Steinheim, Germany GmbH)) following the instructions of the manufacturer. AML-12 cells were homogenized in 300 μl lysis buffer containing protease and phosphatase inhibitors (#P8340, #P5726, #P0044 Sigma-Aldrich Chemie GmbH, Steinheim, Germany). Protein lysate (500 μg) was incubated with an ADH1 antibody (sc-133207; Santa Cruz Biotechnology, TX, USA) and precipitated with Protein A/G Agarose. Precipitated proteins (including a positive control, ADH1 enzyme; #A3263 (Sigma-Aldrich Chemie GmbH, Steinheim, Germany)) were separated on 10 % SDS-polyacrylamide gels and transferred to polyvinylidene difluoride membranes (Bio-Rad Laboratories, Hercules, CA, USA), which were incubated with an anti-phosphoserine antibody (#sc-81514; Santa Cruz Biotechnology, TX, USA) at 4 °C overnight followed by an incubation with a respective secondary antibody (#7076 anti-mouse IgG; #58802 anti-mouse IgG; HRP-linked; Cell Signaling Technology, MA, USA). Protein detection was carried out as detailed above.

### 3.9 RNA isolation, cDNA synthesis and real-time PCR

Total RNA was extracted from liver tissue with TRItidy G™ (VWR International, Vienna, Austria) and cDNA was synthetized (Reverse Transcription System, Promega GmbH, WI, USA) as detailed elsewhere [21]. Real-time polymerase chain reaction (PCR) was performed to determine expression of the respective genes normalized to 18S as previously described [21]. Primer sequences used for real-time PCR are shown in Table S5.

### 3.10 Statistical analysis

All data are presented as means ± standard error of mean (SEM). Statistical analysis was performed using PRISM (version 7.03, GraphPad Software, Inc., MA, USA). Grubb's test was used to determine outliers. Data were log-transformed when not normal distributed or in case of inhomogeneity of variances. A Student t-test was used to analyse differences between C- and SFC-fed animals fed for eight weeks or for analysis between MASLD patients and healthy controls. A one-way ANOVA and two-way ANOVA followed by Tukey's post-hoc test, respectively, were applied to determine statistical differences between three and more different groups. Correlation analysis were performed using Pearson's correlation coefficient. A p-value ≤0.05 was defined as significant.

## 4 Results

### 4.1 Ethanol levels in peripheral blood, ADH activity and protein level in liver tissue of patients with MASLD and non-MASLD patients

Characteristics of MASLD and non-MASLD patients (=controls) undergoing liver resection are provided in Table 1. Patients with MASLD had significantly higher BMI and gamma-glutamyltransferase (γ-GT) levels than those without MASLD while all other parameters including age and aspartate aminotransferase (AST) as well as ALT activity in plasma were similar.

**Fig. 1:**
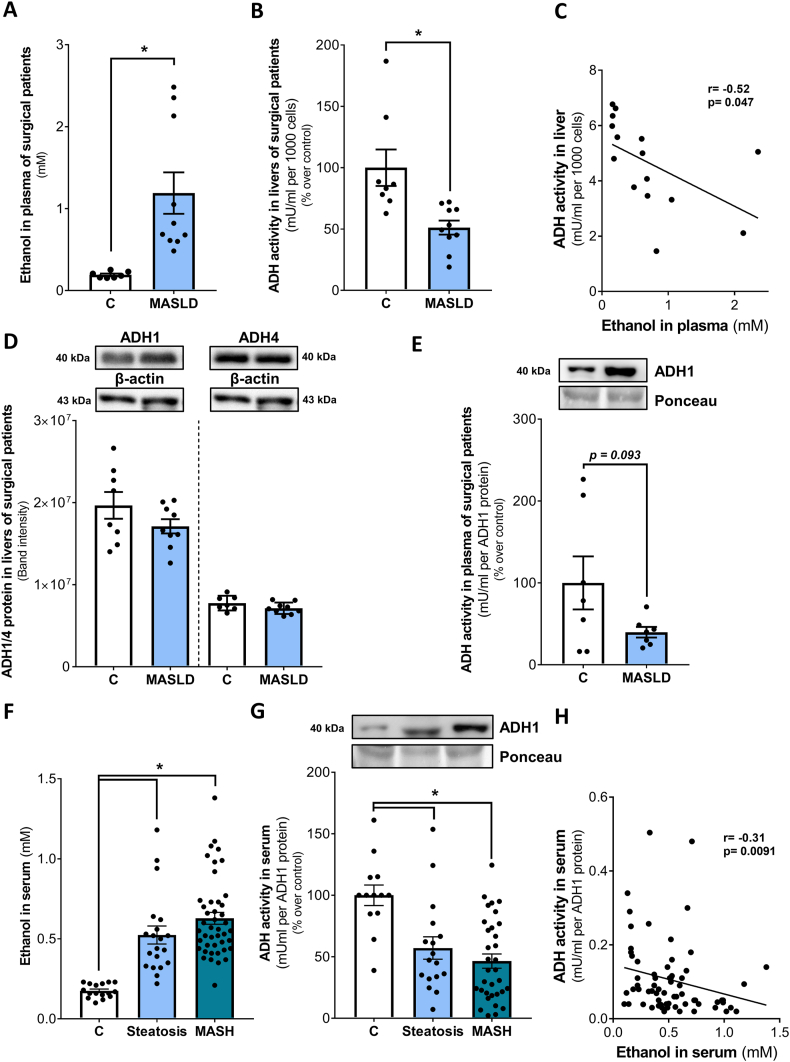
**Ethanol levels and ADH activity in plasma, serum and liver, respectively, of patients with different stages of metabolic dysfunction-associated steatotic liver disease (MASLD) and non-MASLD patients (controls).** (A) Ethanol concentration in plasma, (B) relative ADH activity in the cytosolic fraction of the liver, (C) correlation analysis of ADH activity in liver and ethanol concentration in plasma as well as (D) ADH1 and 4 protein concentration in liver and (E) relative ADH activity in plasma of C^#^ and MASLD patients. (F) Ethanol concentration in serum, (G) relative ADH activity in serum and (H) correlation analysis of ADH activity in serum and ethanol concentration in serum of patients with steatosis, MASH and controls (C). Data are shown as means ± SEM, n = 8 controls^#^ (C^#^), n = 10 MASLD patients (Fig. A-E); n = 16 controls, n = 20 steatosis patients and n = 44 MASH patients (Fig. F-H); *p≤0.05 compared to respective controls calculated by Student's *t*-test or by one-way ANOVA followed by Tukey's post-hoc test or Pearson's correlation coefficient. C^#^, non-MASLD patients undergoing liver resection of medical reasons; C, healthy controls; MASLD, metabolic dysfunction-associated steatotic liver disease; MASH, metabolic dysfunction-associated steatohepatitis; ADH, alcohol dehydrogenase.

Fasting ethanol levels in plasma of MASLD patients undergoing liver resection were significantly higher than those of controls (Fig. 1A) while relative ADH activity in liver was significantly lower in MASLD patients (Fig. 1B). When pooling data and performing a correlation analysis a significant negative association was found between plasma ethanol levels and ADH activity in liver tissue (Fig. 1C). Neither protein levels of ADH1 nor ADH4 being the isoforms of ADH thought to be critical for the metabolism of ethanol (ADH1 at low ethanol concentrations [22,23] and ADH4 at high ethanol concentrations [23,24]) differed between groups (Fig. 1D). Activity and protein levels of ALDH2 shown to mediate the oxidation of acetaldehyde to acetate [24] were similar in livers of MASLD patients and controls (Figs. S1A and B). It has been shown before that ADH activity can also be determined in serum and plasma [25,26]. To determine if the alterations found in liver tissue were also reflected by ADH activity found in serum and plasma, we determined ADH activity normalized to total ADH1 protein being the more prevalent isoform when compared to ADH4 (Fig. 1D). In line with the findings in liver tissue, relative ADH activity was also in trend lower in plasma of patients with MASLD than in controls (p = 0.093) (Fig. 1E). Also, ADH activity in liver tissue was positively associated with relative ADH activity in plasma (Fig. S1C). To verify these findings, we next assessed fasting ethanol levels and relative ADH activity in serum in a second cohort consisting of patients with biopsy proven liver steatosis and MASH as well as healthy controls. Characteristics of patients and controls are provided in Table 2. BMI and activity of ALT and γ-GT in serum were significantly higher in patients with simple steatosis than in controls whereas when comparing MASH patients and controls BMI and activity of AST, ALT and γ-GT in serum were significantly higher. Furthermore, triglyceride and fasting glucose concentration as well as insulin and HOMA-IR were significantly higher in serum of patients with simple steatosis and MASH, respectively, when compared to controls. Fasting ethanol levels in serum were significantly higher in both steatosis and MASH patients than in controls while relative ADH activity in serum was significantly lower in both, steatosis, and MASH patients compared to controls (Fig. 1F and G). In line with the findings in liver tissue, serum ethanol levels were negatively correlated to relative ADH activity in serum (Fig. 1H).

### 4.2 Ethanol levels in small intestine and portal as well as peripheral blood and ADH and ALDH2 activity in male C57BL/6J mice with diet-induced MASLD

**Fig. 2:**
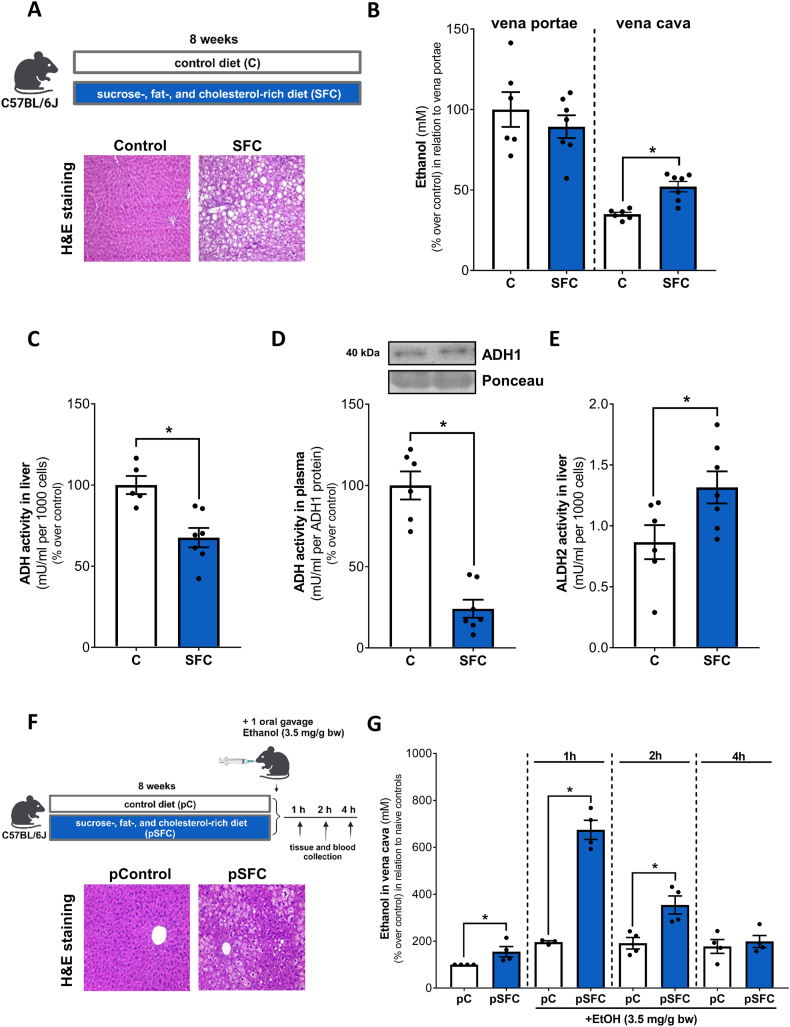
**Effect of the intake of a SFC diet on indices of liver damage and alcohol metabolism in C57BL/6J mice.** (A) Schematic drawing of the animal experiment and representative pictures (magnification 200x) of H&E staining in liver tissue and (B) ethanol concentration in plasma (% over control in relation to vena portae levels) of C- and SFC-fed mice. (C) ADH activity in the cytosolic fraction of the liver, (D) relative ADH activity in plasma and (E) ALDH2 activity in livers of C- and SFC-fed mice. (F) Schematic drawing of the animal experiment and representative pictures (magnification 200x) of H&E stained liver sections of C- and SFC-fed mice and (G) respective ethanol concentrations in plasma (vena cava; % over control in relation to naïve controls) after 1, 2 and 4 h following an alcohol gavage. Data are shown as means ± SEM, n = 6–7 except for (C) n = 5–7; for (G) n = 3–4, *p≤0.05 calculated by Student's t-test. C, control diet; SFC, sucrose-, fat-, and cholesterol-rich diet; ADH, alcohol dehydrogenase; ALDH2, acetaldehyde dehydrogenase 2. Schematic drawings were created with BioRender.com.

Ethanol levels in peripheral blood were significantly higher in mice showing early signs of MASH (e.g., hepatic steatosis with beginning inflammation and insulin resistance, see Fig. 2A and B and Table S1) than in controls. In contrast, ethanol levels neither differed in small intestinal luminal content nor in portal plasma between sucrose-, fat- and cholesterol-rich diet (SFC) and control (C) fed mice (Fig. S1D and Fig. 2B). Also, in line with the findings in humans, total ADH activity was significantly lower in livers of mice showing signs of early MASH (Fig. 2C) while neither ADH1 nor ADH4 protein concentrations differed between groups (Figs. S1E and F). Similar to the findings in liver tissue, relative ADH activity in plasma was also significantly lower in SFC-fed mice than in controls (Fig. 2D). Moreover, ALDH2 protein levels were similar between groups (Fig. S1G), whereas ALDH2 activity was significantly higher in livers of mice with early MASH than in controls (Fig. 2E). Furthermore, ADH activity in liver tissue and relative ADH activity in plasma were significantly positive related (Fig. S1I).

### 4.3 Alcohol elimination in male C57BL/6J mice with diet-induced MASLD

To determine if the impairments of ADH activity found in mice with early signs of MASH affect ethanol elimination, blood alcohol levels were assessed in mice fed a pelleted SFC diet *ad libitum* followed by an alcohol gavage (see Fig. 2F for experimental set-up and Table S2 for characteristics of mice). Basal ethanol levels in peripheral blood of mice showing early signs of MASH were ∼1.5-fold higher than those of naïve controls (p≤0.05). Also, ADH activity in liver tissue was significantly lower in pSFC fed mice than in controls (see Fig. S2A). One hour after ingesting ethanol, plasma ethanol levels in control mice were ∼2-fold higher than in naïve controls. In contrast, in mice with signs of a beginning MASH ethanol levels in plasma were significantly higher than in ethanol exposed control diet fed mice (ethanol exposed pC- vs ethanol exposed pSFC-fed mice +∼3 fold). Two hours after the alcohol ingestion blood ethanol levels were still ∼2-fold higher in plasma of pSFC-fed mice compared to ethanol exposed controls while after 4 h, no differences were observed between groups (Fig. 2G). ADH activity in liver tissue after one and 2 h was significantly lower in pSFC-fed mice than in pC-fed mice, while after 4 h, no differences were observed between groups (Fig. S2A).

### 4.4 Effect of TNFα, IL-1β and IL-6 as well as a JNK agonist and antagonist on ADH activity and phosphorylation in AML-12 cells

**Fig. 3:**
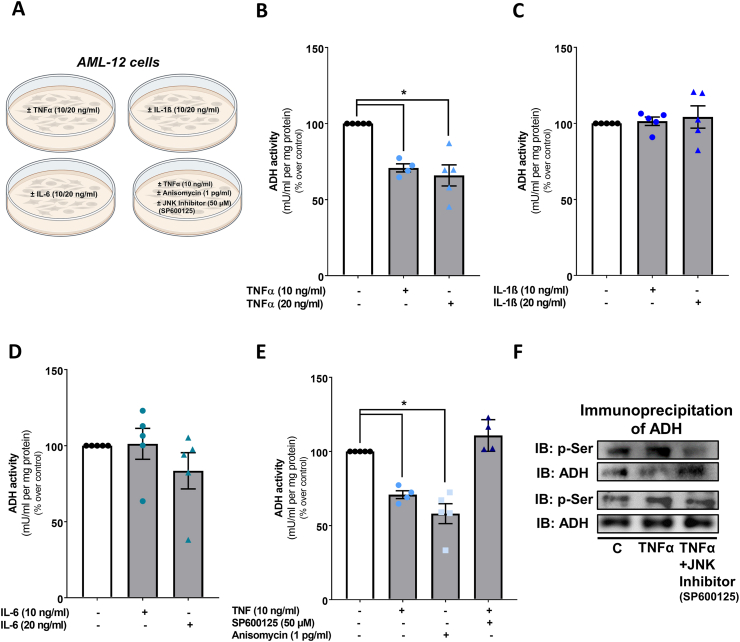
**Effect of a treatment with TNFα, IL-1β and IL-6 as well as a JNK activator and inhibitor on ADH activity in AML-12 cells.** (A) Schematic drawing of the cell culture experiments and ADH activity in cytosolic fraction of AML-12 cells treated with (B) 10 and 20 ng/ml TNFα, (C) 10 and 20 ng/ml IL-1β, (D) 10 and 20 ng/ml IL-6, as well as treated with (E) 50 μM JNK antagonist (SP600125) and 1 pg/ml JNK agonist (anisomycin) (preincubated for 1 h ±10 ng/ml TNFα for 6 h) and (F) representative blots of the ADH immunoprecipitation with 10 ng/ml TNFα and 50 μM SP600125 treated AML-12 cells. Data are shown as means ± SEM, n = 4–5 except for (F) n = 2, *p≤0.05 calculated by one-way ANOVA followed by Tukey's post-hoc test. ADH, alcohol dehydrogenase; anisomycin, JNK agonist; IL-1β, interleukin 1 beta; IL-6, interleukin 6; JNK; c-Jun N-terminal kinase; p-Ser, phospho-Serine; SP600125, JNK antagonist; TNFα, tumor necrosis factor alpha. Schematic drawing was created with BioRender.com.

Next, we challenged AML-12 cells with 10 and 20 ng/ml of TNFα, IL-1β or IL-6, respectively (for experimental set-up see Fig. 3A). In line with our previous findings [4], after being challenged with TNFα for 6 h, ADH activity was significantly decreased by ∼30 % compared to naïve cells (Fig. 3B). In contrast, neither the challenge with 10 or 20 ng/ml of IL-1β nor with similar concentrations of IL-6 affected ADH activity (Fig. 3C and D). As it has been shown by us and others that TNFα may mediate its effect at least in part through an activation of JNK [17,27], we next challenged AML-12 cells with the JNK agonist anisomycin. Suppression of ADH activity induced by anisomycin was almost similar to that found when cells were only challenged with TNFα whereas the concomitant treatment of cells with TNFα and the JNK antagonist SP600125 almost completely attenuated the effects of TNFα on ADH activity (Fig. 3E). Employing immunoprecipitation, we found that in cells challenged with TNFα, serine phosphorylation of ADH was higher than in untreated control cells and that this was attenuated when TNFα challenged cells were pre-incubated with the JNK antagonist SP600125 (Fig. 3F).

### 4.5 Effect of blocking TNFα on ethanol levels in peripheral blood and ADH activity in livers of SFC-fed mice

**Fig. 4:**
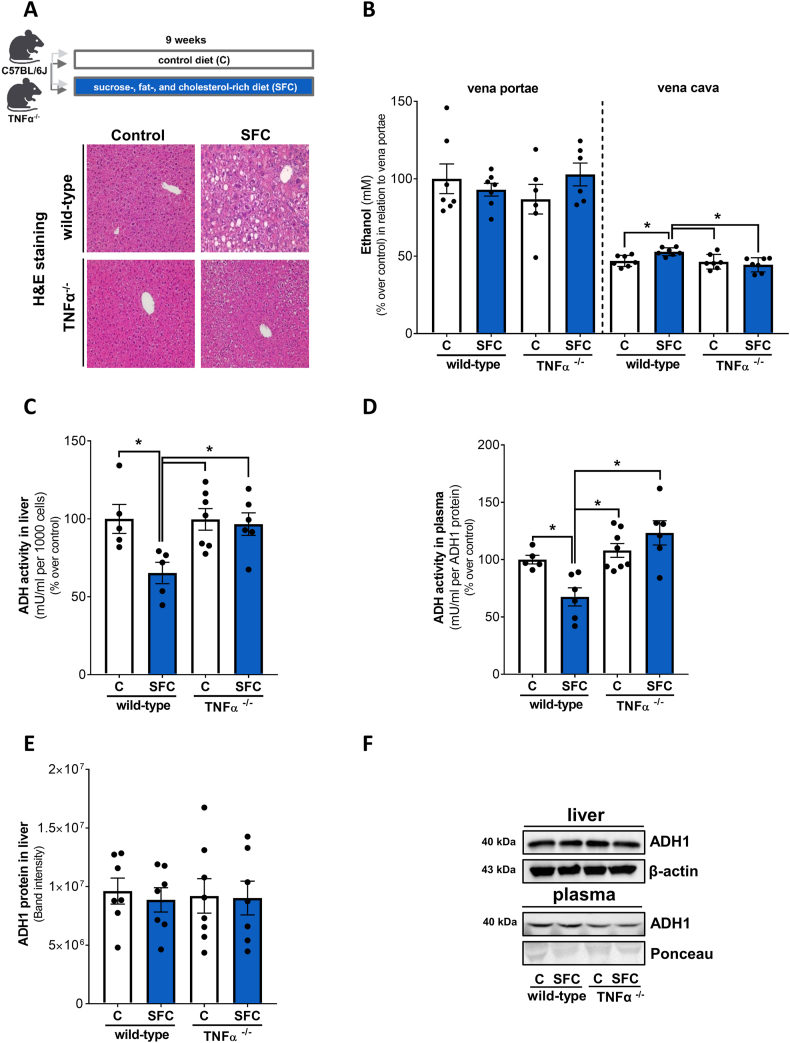
**Effect of the intake of a SFC diet on alcohol metabolism in wild-type and TNFα**^**-/-**^**mice.** (A) Schematic drawing of the animal experiment and representative pictures (magnification 200x) of H&E staining in liver tissue and (B) ethanol concentration in plasma (% over control in relation to vena portae levels), (C) ADH activity in the cytosolic fraction of the liver, (D) relative ADH activity in plasma and (E) ADH1 protein in liver as well as (F) representative blots in liver and plasma. Data are shown as means ± SEM, n = 6–8 except for (C) n = 5–7, for (D) n = 5–8; *p≤0.05 calculated by two-way ANOVA followed by Tukey's post-hoc test. C, control diet; SFC, sucrose-, fat-, and cholesterol-rich diet; ADH, alcohol dehydrogenase; TNFα, tumor necrosis factor alpha. Schematic drawing was created with BioRender.com.

**Fig. 5:**
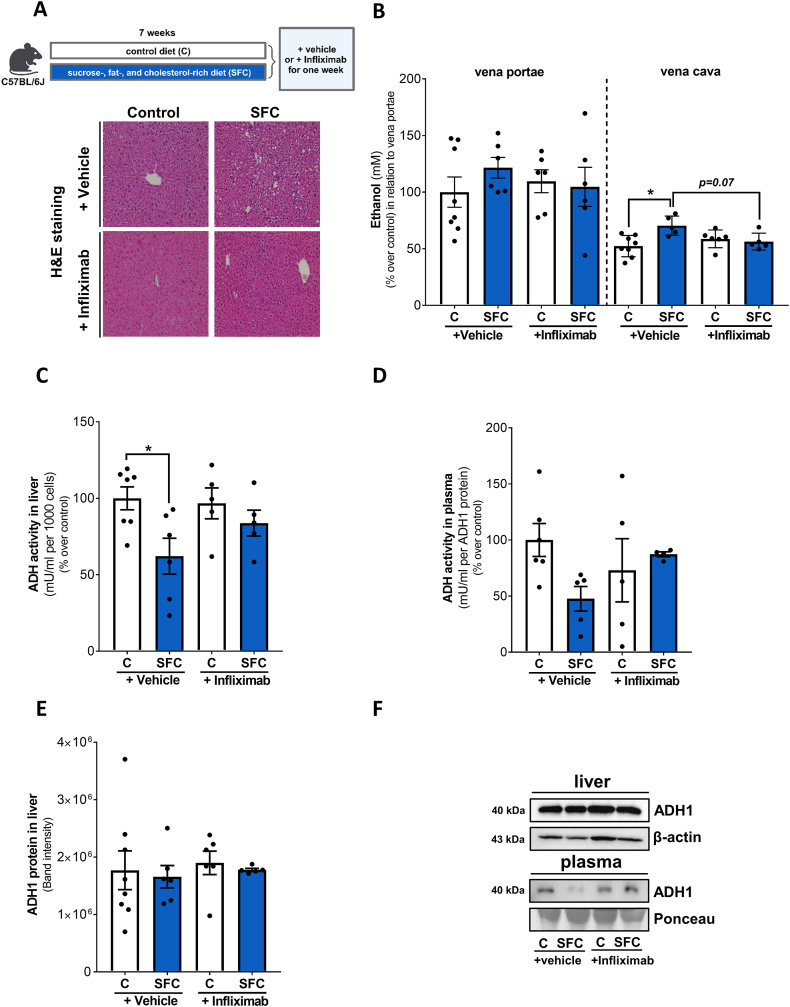
**Effect of the intake of a SFC diet on alcohol metabolism in mice treated with infliximab.** (A) Schematic drawing of the animal experiment and representative pictures (magnification 200x) of H&E staining in liver tissue and (B) ethanol concentration in plasma (% over control in relation to vena portae levels), (C) ADH activity in the cytosolic fraction of the liver, (D) relative ADH activity in plasma and (E) ADH1 protein in liver as well as (F) representative blots in liver and plasma. Data are shown as means ± SEM, n = 6–8 except for (C) n = 5–7, for (D) n = 4–6, for (E) n = 5–8; *p≤0.05 calculated by two-way ANOVA followed by Tukey's post-hoc test. C, control diet; SFC, sucrose-, fat-, and cholesterol-rich diet; ADH, alcohol dehydrogenase. Schematic drawing was created with BioRender.com.

To determine if TNFα is also critical in the regulation of ADH activity and subsequently ethanol levels in peripheral blood *in vivo* in settings of MASLD we assessed ethanol levels in peripheral blood of TNFα^-/-^ mice fed a liquid SFC or a respective control diet. As also reported previously [17] and displayed in Fig. 4A and Table S3, the development of early MASH was significantly attenuated in SFC-fed TNFα^-/-^ mice compared to SFC-fed wild-type animals. While ethanol levels in portal plasma were similar between groups, ethanol levels in peripheral plasma of SFC-fed wild-type mice were significantly higher than in both control groups and in SFC-fed TNFα^-/-^ mice. Ethanol concentration in peripheral blood of SFC-fed TNFα^-/-^ mice was similar to controls (Fig. 4B). ADH activity in liver tissue of SFC-fed wild-type mice was significantly lower than in all other groups whereas ADH activity in livers of SFC-fed TNFα^-/-^ mice was at the level of controls (Fig. 4C). Similar to the findings in liver tissue, relative ADH activity in plasma was also significantly lower in SFC-fed wild-type compared to all other groups (Fig. 4D). In contrast, protein levels of ADH1 in liver tissue were similar in all groups (Fig. 4E and F). In line with these findings, the treatment of mice having a SFC-induced MASLD with infliximab, an anti-TNFα antibody, for one week (see Fig. 5A for experimental set-up and Table S4 for characteristics of mice as well as [17]) resulted in a lowering of peripheral ethanol levels almost to the level of controls (p = 0.07) while ethanol levels in portal plasma were similar between groups (Fig. 5B). Furthermore, the treatment with infliximab in these mice also attenuated the significant decrease in ADH activity in liver tissue found when comparing C-fed mice with SFC-fed animals (Fig. 5C). As data varied considerably, differences alike were not found between groups when assessing relative ADH activity in plasma or ADH1 protein levels in liver tissue (Fig. 5D-F).

## 5 Discussion

MASLD is by now thought to be the most common chronic liver disease world-wide [1,28]. Studies suggest that patients with MASLD but also rodents showing signs of MASLD often have elevated blood ethanol levels even in the absence of any ethanol ingestion [8,9]. Being in line with previous findings [4,8], in the present study, ethanol levels in peripheral blood of patients with early and more progressed stages of the disease and in mice with diet-induced early MASH were elevated. Contrasting the findings of others in humans [10,29] but being in line with previous finding of our own group [4], ethanol levels in intestinal lumen and portal plasma were similar between mice fed the sugar-, fat-, and cholesterol-rich diet and mice fed standard chow. Also, the degree of the difference in ethanol concentration reported for humans in the studies of Meijnikman et al. between portal and peripheral serum (187-fold) [10] was not found in mice in the present study in which we found a reduction by ∼65 % in control mice whereas ethanol levels in MASLD mice were only reduced by ∼37 %. Differences between the results of our studies in mice and others in humans could be related to species differences but also differences in diet and intestinal microbiota composition as well as experimental set-up (mixed meal challenge vs. MASLD- or control diet-fed mice). Indeed, results of our own studies employing liquid diets rather similar to those used in the present study suggest that intestinal microbiota composition in small intestine is only altered by trend but not significantly when animals are fed these diet [30,31] while in the study of Zhu et al. it has been suggested that fecal microbiota composition differed significantly between children with NASH and healthy controls [9]. Furthermore, Meijnikman et al. reported that in small intestine of disease-free overweight subjects and MASLD patients, intestinal microbiota composition only differed by trend but that relative abundance of *Lactobacillaceae* was positively correlated to postprandial ethanol levels [10].

In the present study, the elevation of peripheral ethanol levels was related to lower ADH activities in both liver tissue and peripheral blood of patients with MASLD and mice with MASLD. Moreover, ADH activity in liver and serum of humans was negatively related to ethanol levels in peripheral blood. Furthermore, results of our study also suggest that alcohol clearance after an alcohol binge was markedly delayed in mice with MASLD compared to controls. Studies in rodents of our own group but also those of others have previously suggested that hepatic ADH activity is regulated age-, sex- and bodyweight-specific [32,33] but may also be altered in settings of insulin resistance [4]. It also has been suggested in some studies, that liver status e.g., the presence of cirrhosis [34] but also less severe liver damage may affect alcohol elimination [11]; however, results are rather conflicting which may also be related to the cause of liver disease e.g., chronic alcohol ingestion resulting in an induction of other ethanol metabolizing enzymes like CYP2E1 [35]. Still, while some studies have reported a beneficial effect on MASLD [36,37], results of more recent studies suggest that even moderate alcohol consumption in MASLD patients is related to higher activities of γ-GT and ALT in serum than those found in abstaining MASLD patients [38,39]. If this is also related to impairments of the ADH-dependent elimination of ethanol or other factors e.g., the additional calories concomitantly taken in through the consumption of alcoholic beverages remains to be determined. Moreover, studies suggest that similar to other enzymes like ALT and AST, ADH is released from damaged hepatocytes [40,41]. Results of the present study suggest that relative ADH activity in blood may reflect the findings in liver tissue and that the decrease in ADH activity in livers of patients is reflected by the relative ADH activity in serum.

In summary, our results further bolster the hypothesis that the development and progression of MASLD is associated with elevated blood ethanol levels in peripheral blood even in the absence of any ethanol ingestion and that this is related to impairments of ADH activity, and herein, especially in liver tissue. Furthermore, while in the present study, in mice fed an SFC diet ethanol levels in portal blood were not different from that in controls, our results by no means preclude that ethanol synthesis in the gastrointestinal tract of patients with MASLD is increased. Rather, our results suggest that besides an enhanced ethanol formation in the gastrointestinal tract, alcohol metabolism through ADH may also be impaired thereby adding even further to the increase of blood ethanol levels found in peripheral blood of MASLD patients. Whether these impairments similarly affect alcohol elimination in humans as well as the implications of these impairments on recommendation for alcohol ingestion remains to be determined in future studies. Previous studies of other groups have shown that hepatic ADH activity is regulated age-, sex- and bodyweight-specific [32,33] and that herein insulin-like growth factor I and insulin-dependent signaling pathways may be critical [4,12,42]. Results of previous studies of our own group further suggested that ADH activity may be regulated through TNFα-dependent mechanisms [4]. In line with these findings, in the present study, we showed that TNFα but not IL-1β or IL-6, all found to be induced in the setting of MASLD [17], decreased hepatic ADH activity. We further found that the effect of TNFα on ADH activity was related to a TNFα-dependent activation of JNK and enhanced serine phosphorylation of ADH. It has been shown before that JNK is a key mediator of the intracellular effects of TNFα [17,27]. Furthermore, results of studies in settings of chronic alcohol exposure in rodents suggest that insulin is involved in the regulation of ADH expression [43]; however, in the present study, protein levels of neither ADH1 nor ADH4 were altered in liver tissue in either human or mice with MASLD further supporting our findings, that post-transcriptional measures are involved in the regulation of ADH activity in liver tissue. Also supporting the hypothesis that TNFα is critical in the regulation of ADH activity, peripheral ethanol levels in plasma and ADH activity in liver as well as plasma were similar between TNFα^-/-^ mice fed a MASLD diet and controls. In line with these findings, treatment of mice with MASLD with a TNFα antibody for one week markedly diminished the increase in peripheral plasma ethanol levels and the loss of ADH activity in liver tissue. As liver damage was not fully reverted by the treatment with the antibody, similar effects were not found when assessing relative ADH activity in plasma. Also, it remains to be determined how persistent the inhibition of ADH activity is. It has been shown before that the half-life of ADH protein is 3.5 months [44]. Moreover, studies suggest that ADH activity correlates with ALT and AST activity in serum [40,41], both found to be still elevated in SFC-fed mice treated with infliximab. Notably, anti-TNFα drugs, such as pentoxifylline or infliximab, used in the treatment of alcoholic steatohepatitis have also been tested with various levels of success in the treatment of alcohol-related liver disease in humans [45]. Taken together, our results suggest that impairment of ADH activity in MASLD may be related to an increased serine phosphorylation of the enzyme resulting from an activation of TNFα-dependent signaling cascades and herein especially those related to JNK. Our results further suggest that alterations of ADH activity found in MASLD are reversable and are not related to the expression of the enzyme but rather seem to stem from an allosteric regulation of the enzyme.

## 6 Conclusion

In summary, results of the present study further bolster the hypothesis that the development of MASLD is related to higher peripheral alcohol blood levels even in the absence of any alcohol consumption. Our results also suggest that these increased peripheral alcohol levels may not predominantly result from an increased formation of ethanol in the small or large intestine but rather that ADH-dependent hepatic ethanol clearance may be diminished in settings of MASLD. Furthermore, herein a TNFα-dependent activation of JNK and subsequent serine phosphorylation of ADH seems to be critical. If alcohol clearance is also impaired in patients with MASLD as well as the further mechanistic implications of the impaired ADH activity regarding disease development needs to be determined in future studies.

## CRediT authorship contribution statement

**Katharina Burger:** Writing – review & editing, Writing – original draft, Visualization, Investigation, Formal analysis, Data curation. **Finn Jung:** Investigation, Formal analysis, Data curation. **Katharina Staufer:** Writing – review & editing, Resources. **Ruth Ladurner:** Resources. **Michael Trauner:** Writing – review & editing, Resources. **Anja Baumann:** Writing – review & editing, Investigation, Formal analysis, Data curation. **Annette Brandt:** Writing – review & editing, Investigation, Formal analysis, Data curation. **Ina Bergheim:** Writing – review & editing, Writing – original draft, Visualization, Supervision, Funding acquisition, Formal analysis, Data curation, Conceptualization.

## Declaration of competing interest

KS is an employee of Versantis AG. The other authors disclose no conflicts.

## Data Availability

Data will be made available on request.

## Abbreviations:

ADH: alcohol dehydrogenase
ALDH2: acetaldehyde dehydrogenase 2
ALT: alanine aminotransferase
AST: aspartate aminotransferase
AML-12: alpha mouse liver 12 cells
C: control diet
FBS: fetal bovine serum
GTT: glucose tolerance test
IL: interleukin
i.p.: intraperitoneally
JNK: c-Jun N-terminal kinase
MASH: metabolic dysfunction-associated steatohepatitis
MASLD: metabolic dysfunction-associated steatotic liver disease
NAFLD: non-alcoholic fatty liver disease
NAS: NAFLD activity score
pC: pelleted control diet
SEM: standard error of mean
pSFC: pelleted sucrose-, fat-, and cholesterol-rich diet
SFC: sucrose-, fat-, and cholesterol-rich diet
TLRs: Toll-like receptors
TNFα: tumor necrosis factor alpha
γ-GT: gamma – glutamyltransferase
