# Supplementary material for: MASLD is related to impaired alcohol dehydrogenase (ADH) activity and elevated blood ethanol levels: Role of TNFα and JNK
Source: Redox Biol. 2024 Mar 12;71:103121. doi: 10.1016/j.redox.2024.103121 (PMC10957403; doi:10.1016/j.redox.2024.103121)
Supplement: Multimedia component 1 [file mmc1.pdf]

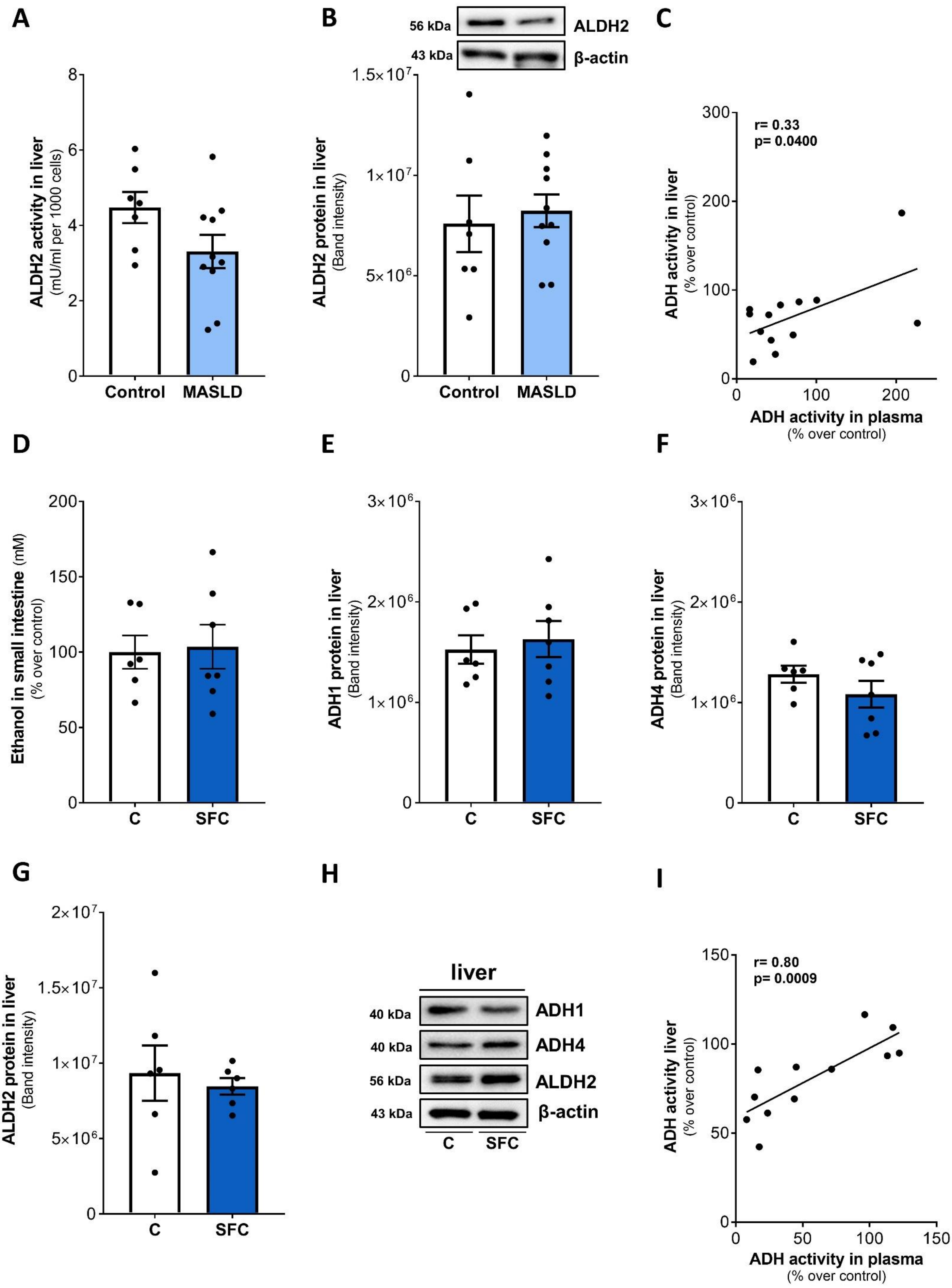
A
ALDH2 activity in liver
(mU/ml per 1000 cells)
Control
MASLD
B
56 kDa
ALDH2
43 kDa
β-actin
ALDH2 protein in liver
(Band intensity)
Control
MASLD
C
r= 0.33
p= 0.0400
ADH activity in liver
(% over control)
ADH activity in plasma
(% over control)
D
Ethanol in small intestine (mM)
(% over control)
C
SFC
E
ADH1 protein in liver
(Band intensity)
C
SFC
F
ADH4 protein in liver
(Band intensity)
C
SFC
G
ALDH2 protein in liver
(Band intensity)
C
SFC
H
liver
40 kDa
ADH1
40 kDa
ADH4
56 kDa
ALDH2
43 kDa
β-actin
C
SFC
I
r= 0.80
p= 0.0009
ADH activity liver
(% over control)
ADH activity in plasma
(% over control)

Supplemental Fig. S1: Markers of alcohol metabolism, correlation of liver and plasma ADH activity in control patients and patients with MASLD as well as control and SFC-fed mice. (A) ALDH2 activity, (B) ALDH2 protein in liver mitochondrial fraction of $C^{\#}$ and MASLD patients and (C) correlation analysis of relative ADH activity in liver and plasma. (D) Ethanol levels in small intestine (% over control) and hepatic protein of (E) ADH1 and (F) ADH4 in liver cytosolic fractions, (G) ALDH2 in liver mitochondrial fractions as well as (H) representative blots and (I) correlation analysis of relative ADH activity in liver and plasma of C- and SFC-fed mice. Data are shown as means ± SEM, n=8 controls[#] ($C^{\#}$), n=10 MASLD patients. For figures D-I n=6-7 except for figure (G) n=6, $^{*}p \leq 0.05$ calculated by Student's t test and Pearson's correlation coefficient (C,I). $C^{\#}$, non-MASLD patients undergoing liver resection of medical reasons, C, control diet; SFC, sucrose-, fat- and cholesterol-rich diet; ADH, alcohol dehydrogenase; ALDH2, acetaldehyde dehydrogenase 2.

A

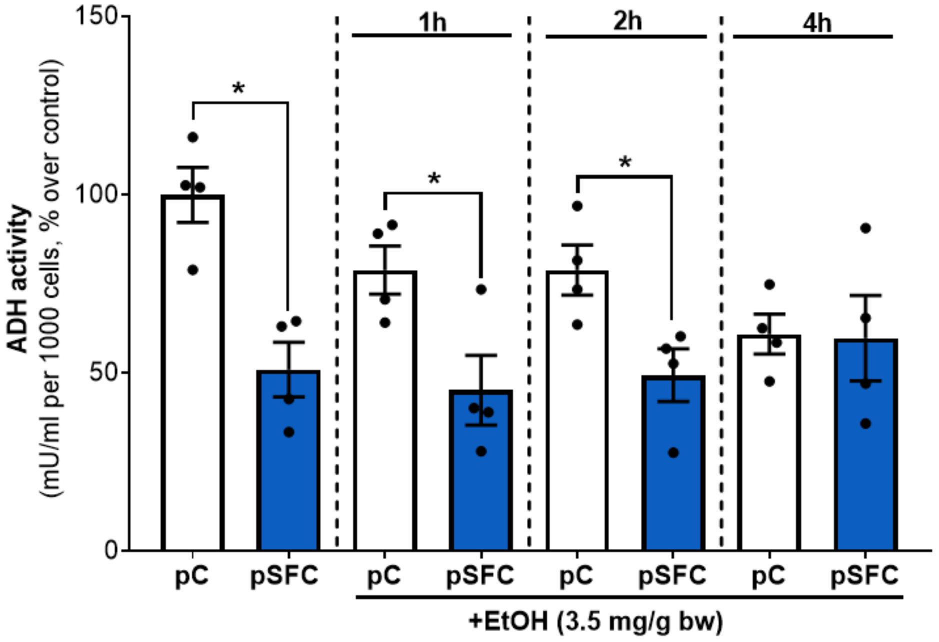

Supplemental Fig. S2: Effect of the intake of a SFC diet on ADH activity in liver tissue of C57BL/6J mice. (A) ADH Activity in the cytosolic fraction of the liver after 1,2 and 4 hours following an alcohol gavage. Data are shown as means ± SEM, n=4, $^{*}p \leq 0.05$ calculated by Student's t-test. C, control diet; SFC, sucrose-, fat- and cholesterol rich diet; ADH, alcohol dehydrogenase.

Table S1: Caloric intake, body and liver weight as well as markers of liver damage and glucose metabolism in SFC-fed C57BL/6J mice.

	Control	SFC
**Caloric intake (kcal/g bw)**	0.40 ± 0.0	0.46 ± 0.0*
**Body weight (g)**	28.2 ± 0.4	29.6 ± 0.7
**Absolute body weight gain (g)**	6.5 ± 0.4	7.0 ± 0.3
**Liver weight (g)**	1.2 ± 0.04	1.6 ± 0.07*
**Liver:body weight ratio (%)**	4.4 ± 0.1	5.3 ± 0.1*
**Steatosis (NAS)**	0.3 ± 0.1	1.8 ± 0.1*
**Inflammation (NAS)**	0.3 ± 0.1	0.7 ± 0.1*
**ALT (U/L)**	7.0 ± 0.9	19.1 ± 3.4*
**Fasting blood glucose (mg/dl)**	103 ± 6	150 ± 5*
**AUC (GTT)**	30,610 ± 1,547	44,010 ± 1,608*
***Tnfa* mRNA expression**[§]	100.0 ± 18.2	193.9 ± 27.2*
***Il6* mRNA expression**[§]	100.0 ± 15.6	189.1 ± 31.6*
***Il1b* mRNA expression**[§]	100.0 ± 13.3	171.6 ± 20.0*

Data are shown as means ± SEM, n=6-8, *p≤0.05 compared to control. [§] mRNA expression in % over control. ALT, alanine aminotransferase; AUC, area under the curve; C, control diet; GTT, glucose tolerance test; *Il1b*, interleukin 1 beta; *Il6*, interleukin 6; NAS, NAFLD activity score; SFC, sucrose-, fat-, and cholesterol-rich diet; *Tnfa*, tumor necrosis factor alpha.

Table S2: Body and liver weight as well as markers of liver damage and glucose metabolism in C57BL/6J mice fed a pelleted SFC or control diet.

	pControl	pSFC
**Body weight (g)**	30.6 ± 0.4	34.4 ± 0.8*
**Absolute body weight gain (g)**	5.9 ± 0.3	9.1 ± 0.6*
**Liver weight (g)**	1.5 ± 0.0	1.7 ± 0.1*
**Liver:body weight ratio (%)**	4.8 ± 0.1	4.9 ± 0.1
**Steatosis (NAS)**	0.0 ± 0.0	1.6 ± 0.3*
**Inflammation (NAS)**	0.3 ± 0.0	0.8 ± 0.2*
**ALT (U/L)**	21.0 ± 2.5	39.4 ± 7.4
**Fasting blood glucose (mg/dl)**	147 ± 4	156 ± 7
**AUC (GTT)**	26,344 ± 1,014	31,504 ± 1,374*

Data are shown as means ± SEM, n=16, *p≤0.05 compared to control-fed mice. ALT, alanine aminotransferase; AUC, area under the curve; pC, pelleted control diet; GTT, glucose tolerance test; NAS, NAFLD activity score; pSFC, pelleted sucrose-, fat-, and cholesterol-rich diet.

Table S3: Caloric intake, body and liver weight as well as markers of liver damage and glucose metabolism in SFC-fed wild-type and TNF$^{-/-}$ mice.

	wild-type mice		TNFα$^{-/-}$ mice	
	**Control**	**SFC**	**Control**	**SFC**
**Caloric intake (kcal/g bw)**	0.39 ± 0.00	0.47 ± 0.01^{a,c}	0.38 ± 0.00	0.46 ± 0.01^{a,c}
**Body weight (g)**	27.7 ± 1.0	29.2 ± 0.7	27.3 ± 0.3	28.1 ± 0.5
**Absolute body weight gain (g)**	3.2 ± 0.7	6.0 ± 0.5^{a}	4.2 ± 0.2	5.5 ± 0.2^{a}
**Liver weight (g)**	1.2 ± 0.1	1.7 ± 0.1^{a,c}	1.3 ± 0.0	1.6 ± 0.0^{a,c}
**Liver:body weight ratio (%)**	4.5 ± 0.1	5.9 ± 0.2^{a,c}	4.6 ± 0.0	5.8 ± 0.1^{a,c}
**Steatosis (NAS)**	0.1 ± 0.0	1.2 ± 0.2^{a,c,d}	0.3 ± 0.1	0.5 ± 0.1^{a}
**Inflammation (NAS)**	0.2 ± 0.1	0.9 ± 0.2^{d}	0.2 ± 0.1	0.4 ± 0.1
**ALT (U/L)**	13.2 ± 1.4	20.6 ± 1.5^{a,c}	15.7 ± 0.7	20.3 ± 1.1^{a,c}
**Fasting blood glucose (mg/dl)**	124 ± 5	165 ± 7^{a,c}	136 ± 6	158 ± 6^{a}
**AUC (GTT)**	28,149 ± 2,463	48,771 ± 3,005^{a,c,d}	32,192 ± 1,642	38,361 ± 2,990^{a}

Data are shown as mean ± SEM, n=6-8; $^{a}p \leq 0.05$ compared to C-fed wild-type mice, $^{c}p \leq 0.05$ compared to C-fed TNFα$^{-/-}$ mice, $^{d}p \leq 0.05$ compared to SFC-fed TNFα$^{-/-}$ mice. Further information regarding study design has been described in detail previously [1]. ALT, alanine aminotransferase; AUC, area under the curve; GTT, glucose tolerance test; NAS, NAFLD activity score; SFC, sucrose-, fat-, and cholesterol-rich diet.

Table S4: Caloric intake, body and liver weight as well as markers of liver damage of C57BL/ 6J mice fed a SFC diet concomitantly treated with infliximab or vehicle.

	Control	SFC	Control	SFC
	+ Vehicle		+ Infliximab	
**Caloric intake (kcal/g bw)**	0.45 ± 0.00	0.43 ± 0.01	0.43 ± 0.01	0.44 ± 0.01
**Body weight (g)**	28.1 ± 0.5	31.4 ± 0.5[a,c]	28.5 ± 0.9	29.4 ± 0.6
**Absolute body weight gain (g)**	5.0 ± 0.7	8.7 ± 0.5[a,c]	4.7 ± 0.6	6.5 ± 0.9
**Liver weight (g)**	1.4 ± 0.0	1.7 ± 0.1[a,c]	1.4 ± 0.1	1.6 ± 0.1
**Liver:body weight ratio (%)**	4.9 ± 0.1	5.2 ± 0.1	5.0 ± 0.1	5.4 ± 0.2
**Steatosis (NAS)**	0.34 ± 0.1	1.83 ± 0.1[a,c]	0.15 ± 0.1	1.38 ± 0.2[a,c]
**Inflammation (NAS)**	0 ± 0	0.46 ± 0.1[a,c,d]	0 ± 0	0.17 ± 0.1[a,c]
**ALT (U/L)**	17.3 ± 2.23	27.2 ± 2.8	24.0 ± 5.3	28.3 ± 3.0

Data are shown as mean ± SEM, n=6-8; [a]$p \leq 0.05$ compared to C-fed + Vehicle-treated mice, [c]$p \leq 0.05$ compared to C-fed + Infliximab-treated mice, [d]$p \leq 0.05$ compared to SFC-fed + Infliximab-treated mice. Further information regarding study design has been described in detail previously [1]. ALT, alanine aminotransferase; NAS, NAFLD activity score; SFC, sucrose-, fat-, and cholesterol-rich diet.

Table S5: Primer sequences used for real-time PCR.

	Forward (5´- 3´)	Reverse (5´- 3´)
18S	GTA ACC CGT TGA ACC CCA TT	CCA TCC AAT CGG TAG TAG CG
Il1b	GTC CGA CAG CAC AGA GGC TTT	TGG CTG TGG AGA AGC TGT GG
Il6	CCA CGC CTT CCC TAC TTC A	TGC AAG TGC ATC ATC GTT GTT C
Tnfa	CAG CCA ACC AGG CAG CGT TCC T	CCT GCC ACA AGC AGG AAT GA

Il1b, interleukin 1 beta; *Il6*, interleukin 6; *Tnfa*, tumor necrosis factor alpha.

References

1. Burger K, Jung F, Baumann A, et al. TNFalpha is a key trigger of inflammation in diet-induced non-obese MASLD in mice. Redox Biol 2023, 66:102870.
